# Checkpoint inhibitor therapy for cancer in solid organ transplantation recipients: an institutional experience and a systematic review of the literature

**DOI:** 10.1186/s40425-019-0585-1

**Published:** 2019-04-16

**Authors:** Noha Abdel-Wahab, Houssein Safa, Ala Abudayyeh, Daniel H. Johnson, Van Anh Trinh, Chrystia M. Zobniw, Heather Lin, Michael K. Wong, Maen Abdelrahim, A. Osama Gaber, Maria E. Suarez-Almazor, Adi Diab

**Affiliations:** 1 Section of Rheumatology and Clinical Immunology, Department of General Internal Medicine, The University of Texas MD Anderson Cancer Center, Houston, TX USA; 2 Department of Rheumatology and Rehabilitation, Faculty of Medicine, Assiut University Hospitals, Assiut, Egypt; 3 Department of Melanoma Medical Oncology, The University of Texas MD Anderson Cancer Center, Houston, TX USA; 4 Section of Nephrology, Division of Internal Medicine, The University of Texas MD Anderson Cancer Center, Houston, TX USA; 5 Department of Biostatistics, The University of Texas MD Anderson Cancer Center, Houston, TX USA; 6 Houston Methodist Hospital, Houston, TX USA

**Keywords:** Checkpoint inhibitors, Cancer, Solid organ transplantation, Alloimmunity

## Abstract

**Background:**

Checkpoint inhibitors (CPIs) have revolutionized the treatment of cancer, but their use remains limited by off-target inflammatory and immune-related adverse events. Solid organ transplantation (SOT) recipients have been excluded from clinical trials owing to concerns about alloimmunity, organ rejection, and immunosuppressive therapy. Thus, we conducted a retrospective study and literature review to evaluate the safety of CPIs in patients with cancer and prior SOT.

**Methods:**

Data were collected from the medical records of patients with cancer and prior SOT who received CPIs at The University of Texas MD Anderson Cancer Center from January 1, 2004, through March 31, 2018. Additionally, we systematically reviewed five databases through April 2018 to identify studies reporting CPIs to treat cancer in SOT recipients. We evaluated the safety of CPIs in terms of alloimmunity, immune-related adverse events, and mortality. We also evaluated tumor response to CPIs.

**Results:**

Thirty-nine patients with allograft transplantation were identified. The median age was 63 years (range 14–79 years), 74% were male, 62% had metastatic melanoma, 77% received anti-PD-1 agents, and 59% had prior renal transplantation, 28% hepatic transplantation, and 13% cardiac transplantation. Median time to CPI initiation after SOT was 9 years (range 0.92–32 years). Allograft rejection occurred in 41% of patients (11/23 renal, 4/11 hepatic, and 1/5 cardiac transplantations), at similar rates for anti-CTLA-4 and anti-PD-1 therapy. The median time to rejection was 21 days (95% confidence interval 19.3–22.8 days). There were no associations between time since SOT and frequency, timing, or type of rejection. Overall, 31% of patients permanently discontinued CPIs because of allograft rejection. Graft loss occurred in 81%, and death was reported in 46%. Of the 12 patients with transplantation biopsies, nine (75%) had acute rejection, and five of these rejections were T cell-mediated. In melanoma patients, 36% responded to CPIs.

**Conclusions:**

SOT recipients had a high allograft rejection rate that was observed shortly after CPI initiation, with high mortality rates. Further studies are needed to optimize the anticancer treatment approach in these patients.

**Electronic supplementary material:**

The online version of this article (10.1186/s40425-019-0585-1) contains supplementary material, which is available to authorized users.

## Introduction

Checkpoint inhibitors (CPIs) have revolutionized the treatment of cancer, with remarkable survival benefits. Since the initial US Food and Drug Administration (FDA) approval of ipilimumab for metastatic melanoma [1], indications for CPIs have expanded into several other cancer types, substantially increasing the number of patients receiving these therapies [2–10]. However, up to 95% of these patients may experience immune-related adverse events (irAEs) [11–15], primarily due to immune dysregulation targeting normal tissue antigens [16, 17].

Safety and efficacy data are lacking for CPIs in patients who have undergone solid organ transplantation (SOT) because these patients have been systematically excluded from clinical trials. However, SOT recipients are known to have an increased risk of developing de novo cancer after SOT [18–22]. Moreover, cancer has been reported as the second leading cause of death in these patients [22], presumably because they receive chronic immunosuppressive therapy to maintain allograft tolerance [23, 24], as well as less aggressive cancer treatments because of comorbidities [25]. As the indications for CPIs expand to many cancers, it is crucial to determine the risk-benefit ratio of CPI use in SOT recipients. In the current study, we reviewed the records of patients who had undergone prior SOT and received CPIs for cancer at The University of Texas MD Anderson Cancer Center. In addition, we systematically reviewed the literature to identify all similar reported patients, to summarize the evidence on the safety of CPIs, including rate of rejection, irAEs, and mortality, and determine the observed tumor response in this population.

## Methods

### Study design

#### Cohort selection

Following institutional review board approval, MD Anderson databases were searched to identify cancer patients who had received CPIs at any time between January 1, 2004, and March 31, 2018. For all patients identified from pharmacy records, claims data from 6 months before the first CPI infusion up to last follow-up or death were extracted. All patients with transplantation claims were identified. International Classification of Diseases 9 and 10 diagnostic codes (V42, V42.0, V42.1, V42.6, V42.7, V42.8, V42.9, V42.83, V42.89, V58.44, 238.77, 996.8, 996.82, 996.84, 996.89, 00.91, 00.93, 33.5, 50.5, 50.51, 50.59, 52.8, 52.80, 55.6, Z48.2, Z48.21, Z94, Z94.0, Z94.1, Z94.2, Z94.3, Z94.4, Z94.8, and Z94.83) were used to identify those with a possible SOT. Medical records with at least one relevant code were reviewed in depth. We included all patients who had a confirmed SOT prior to the initiation of at least one dose of an FDA-approved CPI (ipilimumab, nivolumab, pembrolizumab, atezolizumab, avelumab, or durvalumab).

#### Systematic review

Medline, EMBASE, Web of Science, PubMed ePubs, and the Cochrane Library were searched with no language or study design restrictions through April 4, 2018, to identify studies reporting the use of CPIs in SOT recipients. References in the included articles were searched manually. The search strategy and terms are detailed in Additional file 1. Articles were screened and selected by three independent reviewers (in pairs) using a two-step approach. First, titles and abstracts were reviewed for relevance and inclusion of original data. Then, the full text of the potentially relevant articles was reviewed. Original case reports, case series, and observational studies were included if they described patients with cancer who had a SOT prior to initiation of one of the FDA-approved CPI agents and provided a detailed clinical description of each reported patient. Disagreements between reviewers were resolved by consensus. The quality of the reports was assessed following the recommended guidelines for publishing an adverse event report [26].

#### Outcome assessment

For both patient information obtained from institutional databases and patients identified in the literature, data were extracted by one reviewer and crosschecked by two others. We extracted data on patient demographics and baseline characteristics (type of cancer, type of CPI, prior SOT and its underlying cause, occurrence of allograft rejection prior to CPI initiation, time from SOT to the first CPI infusion, and concomitant immunosuppressive therapy at CPI initiation). We assessed the safety of CPIs in terms of the rate, time to allograft rejection after CPI initiation, and allograft outcome and related mortality. Other typical CPI-induced irAEs were also evaluated. We collected tumor response rates as defined by Response Evaluation Criteria in Solid Tumors 1.1 [27], as well as overall survival (OS), defined as the time from CPI initiation until death for any reason. For patients identified from the literature, most reports did not define the response evaluation criteria used, so we based our determination of the oncologic response to therapy on the authors’ report while remaining cognizant of this limitation.

### Statistical analysis

Data were summarized using descriptive statistics, with medians and interquartile ranges for continuous variables and frequencies and percentages for categorical variables. OS and timing of allograft rejection after CPI initiation were estimated using the Kaplan-Meier method. Data were right-censored at the last available follow-up time at which the patient was known to be alive (for the OS analysis) or free of allograft rejection (for the time to rejection analysis).

## Results

Using MD Anderson databases, we identified 11 patients who met the inclusion criteria; one of them was excluded from the final analysis because of graft loss before CPI initiation. The literature search resulted in 9170 unique citations. Of these, 27 publications described 30 patients who met the inclusion criteria, including a patient who had been identified using the MD Anderson databases. Therefore, 39 patients were included in our final analysis (Additional file 2: Figure S1 and S2).

### Patient characteristics

Patient demographic and baseline characteristics are shown in Table 1. Detailed clinical information for individual patients and the quality appraisal of the cases identified from the literature are summarized in Additional file 3: Table S1 and S2.

**Table 1:** Patient Demographic and Baseline Characteristics (*n* = 39)^a^

Patient characteristic	No. (%)
Median age (range)	63 years (14–79 years)
Sex	
Male	29 (74)
Female	10 (26)
Current cancer	
Metastatic melanoma	24 (62)
Cutaneous squamous cell carcinoma	6 (15)
Non-small cell lung cancer	3 (8)
Hepatocellular carcinoma	4 (10)
Duodenal adenocarcinoma	1 (3)
Malignant peripheral nerve sheath tumor-like melanoma	1 (3)
Checkpoint inhibitor	
Ipilimumab	14 (36)
Anti-PD-1 agents^b^	30 (77)
Nivolumab	14 (36)
Pembrolizumab	17 (44)
Combined use of ipilimumab and nivolumab	1 (3)
Prior solid organ transplantation	
Renal	23 (59)
Hepatic	11 (28)
Cardiac	5 (13)
Allograft rejection before initiation of checkpoint inhibitor therapy (*n* = 24)^c^	4 (17)
Time between transplantation and initiation of checkpoint inhibitor therapy, median (range)	9 years (0.92–32 years)
Pre-emptive modification of the baseline immunosuppressive regimen at initiation of checkpoint inhibitor therapy^d^	20 (51)
Concomitant immunosuppressive therapy at initiation of checkpoint inhibitor therapy^e^	
Corticosteroid	23 (59)
mTOR inhibitor (sirolimus, everolimus)	11 (28)
Calcineurin inhibitor (tacrolimus, cyclosporine)	19 (49)
Other immunosuppressive therapies (azathioprine, mycophenolate mofetil)	6 (15)
No treatment	1 (3)

^a^Abbreviations: mTOR, mechanistic target of rapamycin; anti-PD-1, anti-programmed death-1. Some percentages may not add up to 100 owing to rounding

^b^Six patients switched to anti-PD-1 agents after progression with ipilimumab alone (three switched to pembrolizumab, two switched to nivolumab, and one initially switched to pembrolizumab but was unable to tolerate treatment because of severe constitutional symptoms and later switched to nivolumab)

^c^In 15 patients identified from the literature, it was not reported whether the patient had previous episodes of allograft rejection after transplantation and before the start of checkpoint inhibitor therapy

^d^In two patients, modification of the baseline immunosuppressive regimen was reported before the second checkpoint inhibitor treatment dose

^e^Seventeen patients were receiving combination immunosuppressive therapies at initiation of checkpoint inhibitors

Most patients had metastatic melanoma (62%). Anti-PD-1 agents were used in 30 patients (77%), including six patients who switched to anti-PD-1 agents after their cancer progressed with ipilimumab monotherapy. The melanoma patients were treated per standard of care with anti-PD-1 based regimen as either a first line or salvage therapy after progression on ipilimumab or BRAF inhibitors. All non-melanoma patients received anti-PD-1 agents after they exhausted the respective standard of care regimen including chemotherapies. Prior renal transplantation was reported in 23 patients (59%), hepatic transplantation in 11 patients (28%), and cardiac transplantation in five patients (13%). Median time to initiation of CPI after SOT was 9 years (range 0.92–32 years), and preemptive modification of the baseline immunosuppressive regimen before CPI initiation was reported in 20 patients (51%). Most of the patients received corticosteroids, mTOR inhibitors, calcineurin inhibitors, and/or other immunosuppressive therapies to maintain allograft tolerance.

### Safety

#### Allograft rejection

Overall, 16 patients (41%) had allograft rejection after CPI initiation. The median time to allograft rejection was 21 days (95% confidence interval [CI] 19.3–22.8 days; Table 2). Renal transplantation rejection was reported in 11 patients (48%), hepatic transplantation rejection in four patients (36%), and cardiac transplantation rejection in one patient (20%). Allograft rejection occurred in 2 of the 4 patients (50%) who reported at least one episode of rejection before CPI initiation, and in 9 of the 20 patients (45%) who had no prior rejection. Allograft rejection occurred in 12 of the 30 patients (40%) treated with anti-PD-1 and 5 of the 14 patients (36%) treated with ipilimumab. However, six patients in the anti-PD-1 group had received prior ipilimumab. In 20 patients who had pre-emptive modification of baseline immunosuppressive regimen before CPI initiation, graft rejection occurred in 10 (50%). However, graft rejection also occurred in 6 of the 19 patients (32%) who had no modification of their baseline immunosuppressive therapy. Patients receiving single-agent prednisone (≤10 mg/day) at CPI initiation seemed to have a higher rate of graft rejection, whereas those receiving single-agent calcineurin inhibitors seemed to have the lowest rejection rate (78% compared with 11%; Table 3).

**Table 2:** Checkpoint Inhibitor-Induced Allograft Rejection in Patients with Cancer and Prior Solid Organ Transplantation

Prior organ transplantation	Checkpoint inhibitor	Allograft rejection, no./reported cases (%)	Median time to rejection, days (range)
All		16/39 (41)	15.5 (5–60)
Renal	Ipilimumab	2/4 (50)	21
	Nivolumab	2/5 (40)	18.5 (7–30)
	Pembrolizumab	4/9 (44)	21 (5–60)
	Ipilimumab + nivolumab	1/1 (100)	8
	Ipilimumab followed by nivolumab or pembrolizuamb^a^	2/4 (50)	14.5 (8–21)
	All	11/23 (48)	21 (5–60)
Hepatic	Ipilimumab	1/3 (33)	13
	Nivolumab	2/4 (50)	12.5 (7–18)
	Pembrolizumab	1/3 (33)	7
	Ipilimumab followed by pembrolizumab^a^	0/1 (0)	
	All	4/11 (36)	10 (7–18)
Cardiac	Ipilimumab	0/1 (0)	
	Nivolumab	1/2 (50)	5
	Pembrolizumab	0/1 (0)	
	Ipilimumab followed by pembrolizumab^a^	0/1 (0)	
	All	1/5 (20)	5

^a^Six patients switched to anti-PD-1 agents after progression with ipilimumab alone. For those patients, median time to rejection (when it occurred) was calculated as the time from first infusion of the last checkpoint inhibitor agent until allograft rejection

**Table 3:** Allograft Rejection and Tumor Response Rates According to the Immunosuppressive Regimen Used at Initiation of Checkpoint Inhibitor Therapy

Immunosuppression at initiation of checkpoint inhibitor therapy	Allograft rejection, no./reported cases (%)	Tumor response^a^, no./reported cases (%)
All patients^b^	15/38 (40)	15/32^c^ [47]
Single-agent immunosuppressive therapy		
Prednisone (≤10 mg/day)	7/9 (78)	5/8 (63)
mTOR inhibitors	2/3 (67)	1/2 (50)
Calcineurin inhibitors	1/9 (11)	2/8 (25)
Combination immunosuppressive therapy	5/17 (29)	7/14 (50)

^a^Disease remission or stabilization

^b^One patient stopped anti-rejection medication prior to initiation of ipilimumab + nivolumab combination therapy. This patient had allograft rejection after therapy initiation but re-initiated therapy after a 1-week delay and was able to complete induction therapy, achieving partial tumor response

^c^In six patients, the tumor response to checkpoint inhibitor therapy was not evaluated

There were no differences in the time between SOT and initiation of CPI in patients who had allograft rejection and those who did not. The median time between renal transplantation and CPI initiation was 14 years (range 4.5–25 years) in patients who had rejection and 8 years (range 1.5–32 years) in those who did not have rejection (Additional file 3: Table S1). The time between hepatic transplantation and CPI initiation was ≤5 years in all patients with rejection, but allograft tolerance was reported in a patient who started nivolumab 11 months after SOT [28]. For the only patient who had cardiac transplantation rejection, the time between SOT and CPI initiation was 19 years.

Allograft rejection required treatment with high-dose corticosteroids in 13 of the 16 patients (81%), and sirolimus, tacrolimus, mycophenolate mofetil, or intravenous immunoglobulin were added for five (31%). Dialysis was recommended for 11 patients (69%), 10 with renal transplantation rejection and one with cardiac transplantation rejection, who had reduced ejection fraction with cardio-renal failure requiring temporary dialysis [29]. Despite aggressive treatment, graft loss was observed in 13 patients (81%), 10 with renal transplantation and three with hepatic transplantation. The patient with a rejected cardiac transplantation recovered with residual dysfunction (ejection fraction went down from 55 to 40%) [29]. One patient with renal transplantation and one with hepatic transplantation fully recovered.

#### Immune pathologic features

A transplantation biopsy was performed in 12 of the 16 patients who had rejection. Biopsies suggested acute rejection in nine patients (75%) and complex acute and chronic rejection in the other three. Pathologic features were specified in 10 patients; T cell-mediated rejection was reported in five patients (50%), who initiated CPI therapy at a median of 16 years (range 5–25 years) after SOT. In the other five patients, a combination of cellular- and antibody-mediated rejection was reported, and these patients initiated CPI therapy at a median of 5 years (range 1.9–19 years) after SOT. Immunofluorescence analysis was performed in five patients and showed positive PD-1/PD-L1 expression in four patients and immunoglobulin A deposits in the other patient. C4d immunostaining was tested in four patients and was found positive in one. Donor-specific antibodies were tested in five patients and were found positive in two, but both of them had negative C4d.

#### Immune-related adverse events

In total, eight patients (21%) had CPI-induced irAEs (dermatitis, arthritis, colitis, thyroiditis, hepatitis, pneumonitis, and/or severe constitutional symptoms), all observed in patients who did not have allograft rejection. All irAEs improved with corticosteroids, and mycophenolate mofetil was added for a patient with autoimmune hepatitis [30]. The two patients with thyroiditis were asymptomatic and did not require treatment. Fifteen patients (38%) did not experience either allograft rejection or irAEs. The median duration of follow-up for these patients was 5.5 months (range 2–17 months).

#### Mortality

**Fig. 1:**
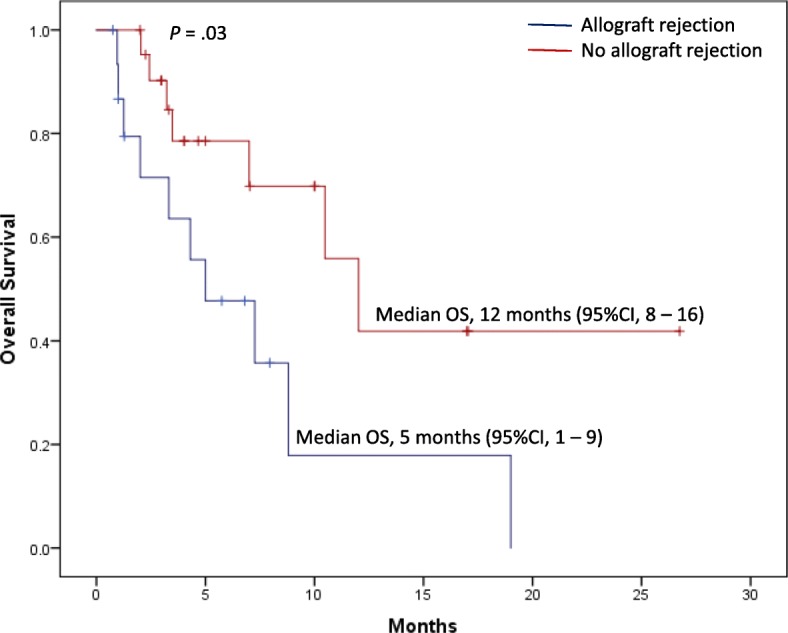
Overall Survival in Patients with and without Allograft Rejection. ^a^In one patient identified from the literature, the overall survival after initiation of the checkpoint inhibitor therapy was not available

Death was reported in 18 patients (46%), primarily because of allograft rejection or rejection complications. This included four renal transplantation recipients (two had synchronous allograft rejection and treatment failure, one had allograft rejection, and one had sudden cardiac death during dialysis after rejection [31]), three hepatic transplantation recipients (two had allograft rejection [32] and one had synchronous allograft rejection and treatment failure), and one cardiac transplantation recipient (synchronous allograft rejection and treatment failure). In the remaining 10 patients, a progressive tumor was reported during anti-rejection immunosuppressive therapy. The median OS in melanoma patients was 10.4 months (95% CI 2.6–18 months). Regardless of cancer type, the median OS was 5 months (95% CI 1–9 months) for patients who had allograft rejection, compared with 12 months (95% CI 8–16 months) for those who had no rejection (*p* = 0.03; Fig. 1). There were no differences in the median OS between patients receiving different immunosuppressive regimens at CPI initiation. The median OS in patients receiving single-agent prednisone (≤10 mg/day) was 5.75 months (95% CI 0.75–19), mTOR inhibitors was 7.26 months (95% CI 1.26–10.02), calcineurin inhibitors was 3.75 months (95% CI 1–26.74), and in those receiving combination immunosuppressive therapies was 3.22 months (95% CI 1–16.99). Although there seems to be numerical differences between the median OS among the four groups favoring the mTOR inhibitors, it is clear that the confidence intervals are overlapping suggesting that there is no statistical significance.

### Tumor response

Tumor responses to CPI therapy for each individual patient are provided in Additional file 3: Table S1. Of the 22 melanoma patients, 14 (64%) had tumor progression and eight (36%) showed favorable tumor responses: seven had complete or partial tumor response and one had stable disease. Tumor responses were numerically less frequent among melanoma patients who had allograft rejection (2/8, 25%) than in those who had no rejection (6/14, 43%). All five patients with cutaneous squamous cell carcinoma achieved complete or partial tumor response. Tumor response was reported in 40% of the patients who had allograft rejection (all cancers combined) and in 52% of those who did not have allograft rejection. Patients receiving single-agent prednisone (≤10 mg/day) at CPI initiation seemed to have numerically higher tumor responses to CPI therapy than those receiving single-agent mTOR inhibitors, calcineurin, or combination immunosuppressant therapy (63% compared with 42%; Table 3**)**. Overall, 12 patients (31%) permanently discontinued CPI therapy because of allograft rejection, and another patient decided to discontinue CPI therapy to avoid allograft rejection with additional treatments [33]. CPIs were temporarily withheld in three patients but successfully re-initiated after stabilization of the patient’s general condition [31, 34, 35]. In addition, three patients discontinued CPI therapy because of other irAEs (hepatitis or pneumonitis), and eight others discontinued CPI therapy because of tumor progression. Twelve patients (31%) were able to continue CPI therapy.

## Discussion

Our data suggest that the use of CPIs in prior SOT recipients may lead to relatively rapid allograft rejection. Allograft rejection occurred in 41% of the patients in our cohort as an adverse event shortly after CPI initiation, and rejection was often accompanied by high mortality rates.

Most patients received anti-PD-1 agents in our cohort, but there was no difference in the frequency of allograft rejection between these patients and those treated with ipilimumab. A few patients switched to anti-PD-1 shortly after progression with ipilimumab; therefore, the possibility of combined dual effects from both classes of CPIs in these patients cannot be completely excluded. The rate of rejection may be higher with PD-1/L1 blockade because alloimmunity largely relies on an alloantigen-mediated response that resembles the mechanism of tumor immune rejection. In addition, anti-PD-1/L1 has higher antitumor response rates than anti-CTLA-4 (CheckMate 067, Keynote 006) [36, 37], and the differing allograft rejection rates may reflect this. In contrast to our finding, anti-CTLA-4 has been previously proposed to be safer than and preferable to anti-PD-1 as the first-line therapy for melanoma in SOT recipients [38]. Because of the small number of patients and the retrospective nature of our study, we cannot definitely infer that the risk of alloimmunity with anti-PD-1 is different from anti-CTLA-4; therefore, prospective studies are needed to definitively answer this question.

We did not observe differences in the frequency of allograft rejection in patients who had a prior episode of rejection before CPI initiation and those who did not. Yet, the small number of patients will not allow reaching definitive conclusion. We also observed differences in the frequency of allograft rejection in patients receiving different immunosuppressive regimens at CPI initiation. Those receiving low-dose prednisone had a higher rejection rate than those receiving other immunosuppressive therapies. However, the data are too scarce to infer which immunosuppressive regimen might have a protective effect against allograft rejection.

Although one could speculate that patients with long-term transplantation would be less prone to rejection when treated with CPIs, we did not find a correlation between the rate and timing of CPI-induced allograft rejection and the time since SOT. Moreover, although T cell-mediated rejection occurs only rarely beyond 10 years after SOT, unlike antibody-mediated rejection [39], we did not find an association between the type of allograft rejection and the time since SOT in our cohort. Transplantation biopsies demonstrated an acute T lymphocyte-mediated rejection process in half of the patients who received CPIs, even at 25 years after SOT. Mixed cellular- and antibody-mediated rejection was observed in the remaining patients. However, the negative C4d immunostaining and donor-specific antibodies in most patients suggest that antibody-mediated rejection did not occur, and this may be a secondary phenomenon induced by the T cell activation that accompanies CPI therapy, triggering a humoral response. The positive expression of PD-1 and PD-L1 proteins in some of the biopsies suggests that the PD-1 pathway contributes to the pathogenesis of allograft tolerance and rejection.

For almost all patients who had rejection, high-dose corticosteroids, and occasionally other aggressive immunosuppressive therapies, along with dialysis and CPI discontinuation, were recommended. Nevertheless, graft loss was observed in 81% of patients. Interestingly, we also observed irAEs similar to those previously reported in phase 3 trials, primarily among patients who did not have rejection. These findings suggest that much of the risk in patients with prior SOT relates to alloimmunity and acute allograft rejection, not necessarily to CPI-induced irAEs. These findings could also suggest a possible difference in the immune mechanisms mediating the occurrence of irAEs and allotransplantation immunity.

The median OS in melanoma patients with prior SOT was much lower than what has been recently reported in the interim analyses of the Keynote-006 trial and the 4-year updated safety analysis of the CheckMate-067 trial [36, 37]. These data suggest that the occurrence of allograft rejection compromised OS in patients with prior SOT. We did not observe significant differences in the OS of patients on each class of immunosuppression. Furthermore, the numbers of patients were too small to infer with confident which immunosuppressive regimen might have compromised OS in this patient population.

Death primarily from allograft rejection or rejection complications was reported in 46% of patients. In regards to tumor response, our data suggest that the concomitant use of anti-rejection therapy at CPI initiation and/or the occurrence of allograft rejection might affect the durability of response to CPI in these patients.

For the effectiveness of CPIs to be maximized in the SOT population, a deep understanding of the molecular and cellular mechanisms underlying both antitumor immunity and alloreactive immunity triggered by CPI administration is a prerequisite to design strategies that can limit harms and fully optimize therapy [40]. Indeed, the functional differences between CTLA-4 and PD-1 inhibitory pathways in allograft transplantation tolerance are not clearly explicit. A much smaller incidence of irAEs with anti-PD-1 agents than with anti-CTLA-4 could give a misguiding notion that anti-PD-1 agents would be safer to use in SOT recipients. However, our data do not support this. Until we have a better understanding of how to uncouple alloreactive immunity from antitumor immunity, treatment in these patients should be focused on the agent with the best anticancer effect. For patients with melanoma, this is anti-PD-1 instead of anti-CTLA-4 therapy.

The use of CPIs in SOT recipients has been previously reported in four other reviews [40–43] and in four case reports with a literature search included [32, 44–46]. However, these reviews did not provide their search strategy or had a more limited search (only one database [40]) and did not follow the methodology required for conducting systematic reviews, such as the screening process and using specific criteria for inclusion and quality assessment. Similar to our findings, the previous reviews identified some patients who had allograft rejection and others who tolerated treatment with no adverse events. However, they did not synthesize the evidence on the frequency of allograft rejection by type of SOT, class of CPI, and type of anti-rejection immunosuppressant used at CPI initiation. The tumor response to CPI and OS in relation to the occurrence of allograft rejection and type of anti-rejection immunosuppression were not clearly specified. In addition, they did not provide information on how rejections were managed and whether they necessitated permanent discontinuation of CPIs.

The largest series to date that retrospectively evaluated the use of CPI therapy in challenging populations included 42 patients with cancer and concurrent diseases; five of these patients were SOT recipients who had received pembrolizumab for concomitant melanoma [47]. Those cases were not included in our analysis because the published results lacked specific information for each patient. No rejection was reported in four patients with renal transplantation, but an acute fatal hepatic rejection occurred immediately after the first treatment dose. These findings suggested that immunotherapy can be given to renal transplantation recipients without rejection. However, the duration of CPI therapy, duration of follow-up for these patients, and whether any of them were receiving anti-rejection immunosuppressive therapy at CPI initiation were not specified. Since our initial search, another retrospective series has been published reporting six renal transplantation recipients who had received ipilimumab for concomitant melanoma [48]. An acute rejection occurred in one patient immediately after the first dose, and safe administration of ipilimumab was observed in the others.

To the best of our knowledge, our study represents the largest single-center cohort of patients with prior SOT receiving CPI therapy. In addition, it is the first systematic review of the literature evaluating the use of CPIs in patients with prior SOT. To put our institutional experience into a meaningful context, we did a systematic search of five databases without restrictions, and with specific criteria for inclusion and for evaluating the quality of reporting in the cases. No prospective observational cohort studies have been published, and therefore, we were not able to ascertain the incidence of allograft rejection in patients with prior SOT. Our data are limited by relying on a retrospective single-center experience and the published case reports, which may not represent the population of interest at large and cannot be used to infer overall frequency. However, our results provide important and much needed safety signals that help clinicians to judiciously weigh the individual risks and benefits for each patient, and our findings generate hypotheses for future studies that may change the treatment paradigm for this patient population.

## Conclusions

The lack of knowledge on the safety and efficacy of CPIs in patients with prior SOT poses a major oncologic challenge. Our data showed a high allograft rejection rate shortly upon CPI initiation that was often accompanied by high mortality rates. At this time, there are no clear recommendations on how to intervene for these patients, hence the risk of allograft rejection should be explicitly conveyed to the patients. Our study highlights the most up-to-date evidence on the use of CPIs in SOT recipients until more robust multi-institutional prospective clinical trials are conducted to evaluate these patients more systematically. Preclinical studies are also needed to enhance our understanding of the complex interactions between the immune system, cancer neoantigens, and alloantigens to establish the ideal therapeutic plan to maintain allograft tolerance and maximize antitumor therapeutic benefits.

## Additional files

Additional file 1: Search Strategy. (DOCX 17 kb)

Additional file 2: **Figure S1.**> MD Anderson Cohort Selection. **Figure S2.** Study Selection Flowchart. (DOCX 48 kb)

Additional file 3: **Table S1.** Immune Checkpoint Inhibitors (CPIs) in Patients with Cancer and Prior Solid Organ Transplantation (SOT)^a^. **Table S2.** Quality Appraisal of the Literature Reported Cases. (DOCX 28 kb)
