# Corrections to: Checkpoint inhibitor therapy for cancer in solid organ transplantation recipients: an institutional experience and a systematic review of the literature

**DOI:** 10.1186/s40425-019-0639-4

**Published:** 2019-06-24

**Authors:** Noha Abdel-Wahab, Houssein Safa, Ala Abudayyeh, Daniel H. Johnson, Van Anh Trinh, Chrystia M. Zobniw, Heather Lin, Michael K. Wong, Maen Abdelrahim, A. Osama Gaber, Maria E. Suarez-Almazor, Adi Diab

**Affiliations:** 1 Section of Rheumatology and Clinical Immunology, Department of General Internal Medicine, The University of Texas MD Anderson Cancer Center, Houston, TX USA; 2 Department of Rheumatology and Rehabilitation, Faculty of Medicine, Assiut University Hospitals, Assiut, Egypt; 3 Department of Melanoma Medical Oncology, The University of Texas MD Anderson Cancer Center, Houston, TX USA; 4 Section of Nephrology, Division of Internal Medicine, The University of Texas MD Anderson Cancer Center, Houston, TX USA; 5 Department of Biostatistics, The University of Texas MD Anderson Cancer Center, Houston, TX USA; 6 Houston Methodist Hospital, Houston, TX USA

**Correction to: J Immunother Cancer (2019) 7:106**

**https://doi.org/10.1186/s40425-019-0585-1**

Following publication of the original article [1], the authors reported an error in the Acknowledgments section. It should be read: ‘We are grateful to Mohsin Shah from the Department of Emergency Medicine at The University of Texas MD Anderson Cancer Center for assisting in study selection, and to Gregory F. Pratt from the Research Medical Library and Erica Goodoff, from the Department of Scientific Publications at The University of Texas MD Anderson Cancer Center for their valuable contributions.
