# Supplementary material for: Checkpoint inhibitor therapy for cancer in solid organ transplantation recipients: an institutional experience and a systematic review of the literature
Source: J Immunother Cancer. 2019 Apr 16;7:106. doi: 10.1186/s40425-019-0585-1 (PMC6469201; doi:10.1186/s40425-019-0585-1)
Supplement: Supplementary file 1 — Search Strategy. (DOCX 17 kb) [file 40425_2019_585_MOESM1_ESM.docx]

**Search Strategy**

Database(s): Ovid MEDLINE In-Process and Other Non-Indexed Citations; Ovid MEDLINE 1946 to 04/03/2018

1 (immun* adj3 checkpoint adj3 (inhibitor* or modulator* or antibod* or block*)).ti,rn.

2 (("cytotoxic T lymphocyte associated" adj3 "4") or "CTLA 4" or CTLA4).ti,rn.

3 (ipilimumab or "MDX CTLA 4" or Yervoy or "MDX 010" or MDX010).ti,rn.

4 (tremelimumab or ticilimumab or "CP 675 206" or "CP 675206" or CP67520).ti,rn.

5 ("Programmed Cell Death 1" or PD1 or "PD 1").ti,rn.

6 (pembrolizumab or Keytruda or Lambrolizumab or "Merck 3475" or Merck3475 or "MK 3475" or MK3475 or "Sch 900475" or Sch900475).ti,rn.

7 (nivolumab or "BMS 936558" or BMS936558 or "MDX 1106" or MDX1106 or "ONO 4538" or ONO4538 or Opdivo).ti,rn.

8 ("AMP 514" or AMP514 or MEDI0680 or "MEDI 0680").ti,rn.

9 ("Programmed Cell Death 2" or PD2 or "PD 2").ti,rn.

10 ("programmed death ligand 1" or "PD L1" or PDL1).ti,rn.

11 (MPDL3280A or "MPDL 3280A").ti,rn.

12 (durvalumab or "MEDI 4736" or MEDI4736).ti,rn.

13 (MSB0010718C or "MSB 0010718C").ti,rn.

14 ("BMS 936559" or BMS936559 or MDX1105 or "MDX 1105").ti,rn.

15 or/1-14

16 exp ANTIBODIES, MONOCLONAL/ae, to

17 (toxic* or safety or (adverse* adj3 (event* or effect*))).ti.

18 exp CHEMICALLY-INDUCED DISORDERS/

19 ADVERSE DRUG REACTION REPORTING SYSTEMS/

20 SAFETY-BASED DRUG WITHDRAWALS/

21 exp TOXICITY TESTS/

22 exp MUTAGENICITY TESTS/

23 CTCAE.ti,ab,kw.

24 ("common terminology criteria" adj3 "adverse events").ti,ab,kw.

25 or/16-24

26 15 and 25 [drug term in ti or rn & toxicity term]

27 (((immun* adj3 checkpoint adj3 (inhibitor* or modulator* or antibod* or block*)) or (("cytotoxic T lymphocyte associated" adj3 "4") or "CTLA 4" or CTLA4) or (ipilimumab or "MDX CTLA 4" or Yervoy or "MDX 010" or MDX010) or (tremelimumab or ticilimumab or "CP 675 206" or "CP 675206" or CP67520) or ("Programmed Cell Death 1" or PD1 or "PD 1") or (pembrolizumab or Keytruda or Lambrolizumab or "Merck 3475" or Merck3475 or "MK 3475" or MK3475 or "Sch 900475" or Sch900475) or (nivolumab or "BMS 936558" or BMS936558 or "MDX 1106" or MDX1106 or "ONO 4538" or ONO4538 or Opdivo) or ("AMP 514" or AMP514 or MEDI0680 or "MEDI 0680") or ("Programmed Cell Death 2" or PD2 or "PD 2") or ("programmed death ligand 1" or "PD L1" or PDL1) or (MPDL3280A or "MPDL 3280A") or (durvalumab or "MEDI 4736" or MEDI4736) or (MSB0010718C or "MSB 0010718C") or ("BMS 936559" or BMS936559 or MDX1105 or "MDX 1105")) adj10 ((adverse* adj5 (event* or effect*)) or toxic* or safety)).ti,ab.

28 26 or 27
