# Supplementary material for: Checkpoint inhibitor therapy for cancer in solid organ transplantation recipients: an institutional experience and a systematic review of the literature
Source: J Immunother Cancer. 2019 Apr 16;7:106. doi: 10.1186/s40425-019-0585-1 (PMC6469201; doi:10.1186/s40425-019-0585-1)
Supplement: Supplementary file 2 — Figure S1.> MD Anderson Cohort Selection. Figure S2. Study Selection Flowchart. (DOCX 48 kb) [file 40425_2019_585_MOESM2_ESM.docx]

**Figure S1.** MD Anderson Cohort Selection

**Patients who received checkpoint inhibitor therapy (n=6,240)**

**Patients with transplantation claims (n=289)**

**(n = 9,168)**

**Patients with stem cell transplantation claims excluded (n = 174)**

- No prior organ transplant

**Patients with organ transplantation claims (n=115)**

**Patients who had confirmed solid organ transplantation prior to treatment initiation (n=9)**

**Two additional patients were identified by the treating physician (n=11)^a^**

^a^Two patients who had received checkpoint inhibitor therapy after March 31, 2018 were identified by the treating physician.

**Figure S2.** Study Selection Flowchart

**Articles identified through bibliography hand-searching (n = 15)**

**Articles identified through databases (n = 13,640)**

- Medline (n = 1,208)
- EMBASE (n = 6,665)
- Web of Science (n = 2,263)
- Cochrane CENTRAL (n = 1,516)
- PubMed ePubs (n = 1,988)

**Unique articles after duplicates (4,485) were removed (n = 9,170)**

**Articles excluded (n = 7,864)**

- Reviews/editorials/notes/comments (n = 2,497)
- Basic science (n = 568)
- Observational studies/trials/case series not reporting individual case descriptions (n = 2,603)
- Not the drug of interest (n = 146)
- Not the population of interest (n = 66)
- Two or more exclusion criteria (n = 1,982)
- Irretrievable (n = 2)

**Full-text articles assessed for eligibility (n = 1,306)**

**Full-text articles excluded (n = 1,279)**

- No prior organ transplantation

**Publications included (n =27)**

**Reported cases (n = 30)**
