# Supplementary material for: Checkpoint inhibitor therapy for cancer in solid organ transplantation recipients: an institutional experience and a systematic review of the literature
Source: J Immunother Cancer. 2019 Apr 16;7:106. doi: 10.1186/s40425-019-0585-1 (PMC6469201; doi:10.1186/s40425-019-0585-1)
Supplement: Supplementary file 3 — Table S1. Immune Checkpoint Inhibitors (CPIs) in Patients with Cancer and Prior Solid Organ Transplantation (SOT)a. Table S2. Quality Appraisal of the Literature Reported Cases. (DOCX 28 kb) [file 40425_2019_585_MOESM3_ESM.docx]

**Table S1.** Immune Checkpoint Inhibitors (CPIs) in Patients with Cancer and Prior Solid Organ Transplantation (SOT)^a^

| **Patient ID**  **(age, sex)** | **Type of cancer** | **Type of CPI** | **Type of SOT (underlying cause)** | **Time from SOT to CPI, years** | **Immunosuppression at CPI initiation (dose/day)** | **CPI-induced adverse events** | **Tumor response to CPI** | **Mortality (cause of death)** |
| --- | --- | --- | --- | --- | --- | --- | --- | --- |
| **Patients with renal transplantation** | | | | | | | | |
| 1 (67, M)^b^ | Melanoma | Ipilimumab | Kidney (PKD) | 19 | Prednisone (5 mg) + cyclosporine (100 mg) | Rejection | PD | Yes (progressive cancer/graft rejection) |
| 2 (40, M)(1) | Melanoma (choroidal) | Ipilimumab | Kidney (Undefined) | 16 | Prednisone (5 mg) | Rejection | PD | Yes (progressive cancer) |
| 3 (63, F)(2) | Melanoma | Nivolumab | Kidney (Type I DM, HTN) | 12 | Prednisone (10 mg) | Rejection | PR | No |
| 4 (48, M)(3) | Melanoma | Nivolumab^c^ | Kidney (IgA nephropathy) | 14 | Prednisone (5 mg) | Rejection | PR | No |
| 5 (60, M)^b^ | Melanoma | Pembrolizumab | Kidney (HTN) | 5 | Prednisone (5 mg) | Rejection | PD | Yes (graft rejection) |
| 6 (58, M)(4) | Melanoma | Pembrolizumab | Kidney (IgM nephropathy) | 13 | Azathioprine (100 mg) + everolimus (1 mg) | Rejection | PD | Yes (progressive cancer/graft rejection) |
| 7 (72, M)^b^ | Melanoma | Pembrolizumab | Kidney (PKD) | 19 | Prednisone (5 mg) + Cyclosporine (175 mg) + MMF (1000 mg)^d^ | Rejection | Not evaluated | No |
| 8 (68, M)(5) | Melanoma | Pembrolizumab^c^ | Kidney (PKD) | 15 | Prednisone (unspecified dose) | Rejection | Undefined | No |
| 9 (72, M)(6) | Cut. SCC | Ipilimumab + nivolumab combination | Kidney (DM) | 4.5 | CPI started after 1 week of immunosuppression (MMF + sirolimus) washout period | Rejection | PR | Yes (rejection complication)^e^ |
| 10 (57, F)(7) | Cut. SCC | Pembrolizumab | Kidney (undefined) | 25 | Prednisone (5 mg) | Rejection | PR | No |
| 11 (74, M)(8) | NSCLC | Nivolumab | Kidney (DM, HTN) | 5 | Prednisone + cyclosporine (unspecified doses) | Rejection | Undefined | No |
| 12 (58, M)(9) | Melanoma | Ipilimumab | Kidney (PKD) | 8 | Prednisone (5 mg) | Colitis | PR | No |
| 13 (79, M)^b^ | Melanoma | Nivolumab^c^ | Kidney (HTN, FSGS) | 4 | Prednisone (2.5 mg) + cyclosporine (200 mg) | Arthralgia, severe constitutional symptoms | PR | No |
| 14 (71, M)^b^ | Melanoma | Pembrolizumab | Kidney (DM) | 2 | Prednisone (5 mg) + sirolimus (3 mg) | Dermatitis, hepatitis, severe constitutional symptoms | PD | Yes (progressive cancer) |
| 15 (66, M)^b^ | Melanoma | Pembrolizumab | Kidney (RCC) | 1.5 | Prednisone (5 mg) + MMF (1000 mg) + tacrolimus (3 mg) | Thyroiditis | PD | Yes (progressive cancer) |
| 16 (69, F)(10) | Cut. SCC | Nivolumab | Kidney (FSGS) | 14 | Prednisone (5 mg) + sirolimus (unspecified) | Dermatitis, pneumonitis | PR | No |
| 17 (72, M)(9) | Melanoma | Ipilimumab | Kidney (HTN) | 11 | Prednisone (5 mg) | None | PR | No |
| 18 (60, F)(11) | Melanoma | Nivolumab | Kidney (PKD) | 13 | Prednisolone + MMF (unspecified doses) | None | PD | Yes (progressive cancer) |
| 19 (77, M)(12) | Melanoma | Nivolumab^c^ | Kidney (DM) | 8 | Prednisone (5 mg) + tacrolimus (4 mg) | None | PD | No |
| 20 (58, M)(11) | Melanoma (Uveal) | Pembrolizumab | Kidney (Hydronephrosis) | 32 | Cyclosporine (unspecified dose) | None | PD | Yes (progressive cancer) |
| 21 (63, M)(13) | Cut. SCC | Pembrolizumab | Kidney (nephrosclerosis) | 13 | Prednisone (2.5 mg) + sirolimus (2 mg) | None | CR | No |
| 22 (61, M)^b^ | Cut. SCC | Pembrolizumab | Kidney (IgA nephropathy) | 4 | Prednisone (5 mg) + Tacrolimus (2 mg)^f^ | None | PR | No |
| 23 (70, M)(14) | Duodenal cancer | Nivolumab | Kidney (RCC) | 6 | Prednisone (40 mg) + sirolimus (unspecified) | None | SD | No |
| **Patients with hepatic transplantation** | | | | | | | | |
| 24 (67, F)(15) | Melanoma (ocular) | Ipilimumab | Liver (metastasis) | 1.5 | Prednisolone (10 mg) | Rejection | PD | Yes (progressive cancer) |
| 25 (46, F)^b^ | Melanoma | Pembrolizumab^g^ | Liver (medication toxicity) | 5 | Sirolimus (5 mg) | Rejection | PD | Yes (progressive cancer/graft rejection) |
| 26 (20, M)(16, 17) | HCC | Nivolumab | Liver | 3.3 | Sirolimus (unspecified dose) | Rejection | Undefined | Yes (graft rejection) |
| 27 (14, M)(16, 17) | HCC | Nivolumab | Liver | 1.9 | Tacrolimus (unspecified dose) | Rejection | Undefined | Yes (graft rejection) |
| 28 (67, M)(18) | Melanoma | Ipilimumab | Liver (HCV, HCC) | 8 | Sirolimus (1 mg) | Dermatitis, hepatitis | PR | No |
| 29 (35, M)(19)^b^ | Melanoma | Pembrolizumab | Liver (biliary atresia) | 20 | Tacrolimus (2 mg) | Hepatitis | CR | No |
| 30 (59, F)(20) | Melanoma | Ipilimumab | Liver (alpha 1 antitrypsin deficiency cirrhosis) | 8 | Tacrolimus (2 mg) | None | PD | No |
| 31 (41, M)(21) | HCC | Nivolumab | Liver (HCV, HIV, liver cirrhosis, HCC) | 0.92 | Tacrolimus (1 mg) | None | PD | Yes (progressive cancer) |
| 32 (70, M)(22) | HCC | Pembrolizumab | Liver (cryptogenic cirrhosis, HCC) | 8 | Tacrolimus (unspecified dose) | None | PD | Yes (progressive cancer) |
| 33 (62, F)(23) | MPNST-like melanoma | Pembrolizumab^c^ | Liver (alcoholic cirrhosis, HCC) | 6 | MMF (1000 mg) + sirolimus (1 mg) | None | PR | No |
| 34 (54, M)(24) | NSCLC | Nivolumab | Liver (HCV) | 13 | Prednisone (60 mg) + tacrolimus (0.5 mg) + everolimus (2 mg) | None | PD | Yes (progressive cancer) |
| **Patients with cardiac transplantation** | | | | | | | | |
| 35 (49, M)(25) | Cut. SCC | Nivolumab | Cardiac  (familial dilated cardiomyopathy) | 19 | Prednisone + tacrolimus + sirolimus (unspecified doses) | Rejection | Not evaluated | Yes (progressive cancer/graft rejection) |
| 36 (69, F)(26) | Melanoma | Ipilimumab | Cardiac (undefined) | 15 | Tacrolimus (2.5 mg) | None | SD | Yes (progressive cancer) |
| 37 (73, M)^b^ | Melanoma | Pembrolizumab | Cardiac (dilated cardiomyopathy) | 9 | Tacrolimus (6 mg) | None | PD | No |
| 38 (75, M)(27) | Melanoma | Pembrolizumab^c^ | Cardiac (ischemic cardiomyopathy) | 12 | Tacrolimus (6 mg) | None | PD | No |
| 39 (72, F)(10) | NSCLC | Nivolumab | Cardiac (RHD) | 10 | MMF + cyclosporine (unspecified doses) | None | SD | No |
|  |  |  |  |  |  |  |  |  |

^a^M, male; F, female; HTN, hypertension; PD, progressive disease; PKD, polycystic kidney disease; DM, diabetes mellitus; PR, partial response; NSCLC, non-small cell lung cancer; Cut. SCC, cutaneous squamous cell carcinoma; MMF, mycophenolate mofetil; FSGS, focal segmental glomerulosclerosis; RCC, renal cell carcinoma; SD, stable disease; CR, complete response; HCC, hepatocellular carcinoma; HCV, hepatitis C virus; MPNST, malignant peripheral nerve sheath tumor; HIV, human immunodeficiency virus; RHD, rheumatic heart disease.

^b^MD Anderson patients (n = 10).

^c^Previous use of ipilimumab was reported in these patients, and patient 13 had previously received both ipilimumab and pembrolizumab.

^d^One week before the second dose, MMF was discontinued, cyclosporine dose decreased to 150 mg and prednisone dose left unchanged.

^e^The patient had CPI-induced graft loss and underwent dialysis. Five months later, the patient experienced sudden cardiac death of unclear etiology during dialysis.

^f^Tacrolimus was being titrated progressively before CPI initiation and was stopped after the first dose.

^g^Previous use of interleukin-2 was reported 2 weeks before initiation of pembrolizumab and was stopped because of liver toxicity.

**Table S2.** Quality Appraisal of the Literature Reported Cases.

| **Lead author (citation)** | **Title** | **Patient demographics** | **Current health status** | **Medical history** | **Physical exam** | **Patient disposition** | **Drug identification** | **Dosage** | **Drug reaction interface** | **Concomitant therapy** | **Adverse events** | **Discussion** |
| --- | --- | --- | --- | --- | --- | --- | --- | --- | --- | --- | --- | --- |
| Alhamad(5) | Agree | Agree | Agree | Agree | Agree | Agree | Agree | Agree | Agree | Agree | Agree | Partially agree |
| Barnett(14) | Agree | Agree | Agree | Agree | Agree | Agree | Agree | Agree | Agree | Agree | Agree | Agree |
| Biondani(24) | Agree | Agree | Agree | Agree | Agree | Agree | Agree | Agree | Agree | Agree | Agree | Agree |
| Boils(8) | Agree | Agree | Agree | Agree | Agree | Agree | Agree | Partially agree | Agree | Agree | Agree | Partially agree |
| De Toni(21) | Agree | Agree | Agree | Agree | Agree | Agree | Agree | Agree | Agree | Agree | Agree | Partially agree |
| Dueland(15) | Agree | Agree | Agree | Agree | Agree | Agree | Agree | Agree | Agree | Agree | Agree | Agree |
| Friend(16, 17)^b^ | Agree | Agree | Agree | Agree | Agree | Agree | Agree | Agree | Agree | Agree | Agree | Agree |
|  | Agree | Agree | Agree | Agree | Agree | Agree | Agree | Agree | Agree | Agree | Agree | Agree |
| Gastman(26) | Agree | Agree | Agree | Agree | Agree | Agree | Agree | Agree | Agree | Agree | Agree | Agree |
| Herz(12) | Agree | Agree | Agree | Agree | Agree | Agree | Agree | Agree | Agree | Agree | Agree | Agree |
| Jose(1) | Agree | Agree | Agree | Agree | Agree | Agree | Agree | Agree | Agree | Agree | Agree | Agree |
| Kittai(10) | Agree | Agree | Agree | Agree | Agree | Agree | Agree | Partially agree | Agree | Agree | Agree | Agree |
|  | Agree | Agree | Agree | Agree | Agree | Agree | Agree | Partially agree | Agree | Agree | Agree | Agree |
| Kuo(23) | Agree | Agree | Agree | Agree | Agree | Agree | Agree | Agree | Agree | Agree | Agree | Agree |
| Kwatra(4) | Agree | Agree | Agree | Agree | Agree | Agree | Agree | Agree | Agree | Agree | Agree | Agree |
| Lipson(9) | Agree | Agree | Agree | Agree | Agree | Agree | Agree | Partially agree | Agree | Agree | Agree | Agree |
|  | Agree | Agree | Agree | Agree | Agree | Agree | Agree | Partially agree | Agree | Agree | Agree | Agree |
| Lipson(7) | Agree | Agree | Agree | Agree | Agree | Agree | Agree | Partially agree | Agree | Agree | Agree | Partially agree |
| Miller(6) | Agree | Agree | Agree | Agree | Agree | Agree | Agree | Partially agree | Agree | Agree | Agree | Agree |
| Morales(18) | Agree | Agree | Agree | Agree | Agree | Agree | Agree | Agree | Agree | Agree | Agree | Agree |
| Ong(2) | Agree | Agree | Agree | Agree | Agree | Agree | Agree | Agree | Agree | Agree | Agree | Agree |
| Owonikoko (25) | Agree | Agree | Agree | Agree | Agree | Agree | Agree | Partially agree | Agree | Agree | Agree | Agree |
| Qin(27) | Agree | Agree | Agree | Agree | Agree | Agree | Agree | Agree | Agree | Agree | Agree | Agree |
| Ranganath(20) | Agree | Agree | Agree | Agree | Agree | Agree | Agree | Agree | Agree | Agree | Agree | Agree |
| Sadaat(13) | Agree | Agree | Agree | Agree | Agree | Agree | Agree | Agree | Agree | Agree | Agree | Agree |
| Schvartsman(19) | Agree | Agree | Agree | Agree | Agree | Agree | Agree | Agree | Agree | Agree | Agree | Partially agree |
| Spain(3) | Agree | Agree | Agree | Agree | Agree | Agree | Agree | Agree | Agree | Agree | Agree | Agree |
| Varkaris(22) | Agree | Agree | Agree | Agree | Agree | Agree | Agree | Partially agree | Agree | Agree | Agree | Agree |
| Winkler(11) | Agree | Agree | Agree | Agree | Agree | Agree | Agree | Partially agree | Agree | Agree | Agree | Agree |
|  | Agree | Agree | Agree | Agree | Agree | Agree | Agree | Partially agree | Agree | Agree | Agree | Agree |

^a^The quality of reporting the cases identified from the literature was evaluated by one reviewer (HS) and cross-checked by another (NA) based on the 12 elements required by the International Society for Pharmacoepidemiology and the International Society of Pharmacovigilance for publishing adverse events reports. Possible item ratings are agree, partially, or disagree.

^b^Two publications for the same case reports.

**REFERENCES**

1. Jose A, Yiannoullou P, Bhutani S, Denley H, Morton M, Picton M, et al. Renal allograft failure after ipilimumab therapy for metastatic melanoma: a case report and review of the literature. Transplant Proc. 2016;48(9):3137-41.

2. Ong M, Ibrahim AM, Bourassa-Blanchette S, Canil C, Fairhead T, Knoll G. Antitumor activity of nivolumab on hemodialysis after renal allograft rejection. Journal for ImmunoTherapy of Cancer. 2016;4 (1) (no pagination)(64).

3. Spain L, Higgins R, Gopalakrishnan K, Turajlic S, Gore M, Larkin J. Acute renal allograft rejection after immune checkpoint inhibitor therapy for metastatic melanoma. Annals of Oncology. 2016;27(6):1135-7.

4. Kwatra V, Karanth NV, Priyadarshana K, Charakidis M. Pembrolizumab for metastatic melanoma in a renal allograft recipient with subsequent graft rejection and treatment response failure: a case report. Journal of Medical Case Reports. 2017;11 (1) (no pagination)(79).

5. Alhamad T, Venkatachalam K, Linette GP, Brennan DC. Checkpoint inhibitors in kidney transplant recipients and the potential risk of rejection. American journal of transplantation : official journal of the American Society of Transplantation and the American Society of Transplant Surgeons. 2016;16(4):1332-3.

6. Miller DM, Faulkner-Jones BE, Stone JR, Drews RE. Complete pathologic response of metastatic cutaneous squamous cell carcinoma and allograft rejection after treatment with combination immune checkpoint blockade. JAAD Case Reports. 2017;3(5):412-5.

7. Lipson EJ, Bagnasco SM, Moore J, Jang S, Patel MJ, Zachary AA, et al. Tumor regression and allograft rejection after administration of anti-PD-1. New England Journal of Medicine. 2016;374(9):896-8.

8. Boils CL, Aljadir DN, Cantafio AW. Use of the PD-1 pathway inhibitor nivolumab in a renal transplant patient with malignancy. American Journal of Transplantation. 2016;16(8):2496-7.

9. Lipson EJ, Bodell MA, Kraus ES, Sharfman WH. Successful administration of ipilimumab to two kidney transplantation patients with metastatic melanoma. Journal of Clinical Oncology. 2014;32(19):e69-e71.

10. Kittai AS, Oldham H, Cetnar J, Taylor M. Immune checkpoint inhibitors in organ transplant patients. Journal of Immunotherapy. 2017;40(7):277-81.

11. Winkler JK, Gutzmer R, Bender C, Lang N, Zeier M, Enk AH, et al. Safe administration of an anti-PD-1 antibody to kidney-transplant patients: 2 clinical cases and review of the literature. Journal of immunotherapy. 2017;40(9):341-4.

12. Herz S, Hofer T, Papapanagiotou M, Leyh JC, Meyenburg S, Schadendorf D, et al. Checkpoint inhibitors in chronic kidney failure and an organ transplant recipient. European Journal of Cancer. 2016;67:66-72.

13. Sadaat M, Jang S. Complete tumor response to pembrolizumab and allograft preservation in renal allograft recipient on immunosuppressive therapy. Journal of Oncology Practice. 2018;14(3):198-9.

14. Barnett R, Barta VS, Jhaveri KD. Preserved renal-allograft function and the PD-1 pathway inhibitor nivolumab: To the editor. New England Journal of Medicine. 2017;376(2):191-2.

15. Dueland S, Guren TK, Boberg KM, Reims HM, Grzyb K, Aamdal S, et al. Acute liver graft rejection after ipilimumab therapy. Annals of Oncology. 2017;28(10):2619-20.

16. Friend BD, Venick RS, McDiarmid SV, Zhou X, Naini B, Wang H, et al. Fatal orthotopic liver transplant organ rejection induced by a checkpoint inhibitor in two patients with refractory, metastatic hepatocellular carcinoma. Pediatr Blood Cancer. 2017;64(12).

17. Zhou X, Naini BV, Federman NC, Garcia-Tsao G, Marcus EA, Venick RS, et al. Liver injury induced by PD-1 inhibitor nivolumab: Report of 3 cases with biopsy findings. Laboratory Investigation. 2017;97:427A.

18. Morales RE, Shoushtari AN, Walsh MM, Grewal P, Lipson EJ, Carvajal RD. Safety and efficacy of ipilimumab to treat advanced melanoma in the setting of liver transplantation. Journal for Immunotherapy of Cancer. 2015;3:22.

19. Schvartsman GP, K. Sood, G. Katkhuda, R. Tawbi, H. Immune checkpoint inhibitor therapy in a liver transplant recipient with melanoma. Annals of internal medicine. 2017;167(5):361-2.

20. Ranganath HA, Panella TJ. Administration of ipilimumab to a liver transplant recipient with unresectable metastatic melanoma. Journal of immunotherapy. 2015;38(5):211.

21. De Toni ENG, A. L. Tapering of immunosuppression and sustained treatment with nivolumab in a liver transplant recipient. Gastroenterology. 2017;152(6):1631-3.

22. Varkaris A, Lewis DW, Nugent FW. Preserved liver transplant after PD-1 pathway inhibitor for hepatocellular carcinoma. Am J Gastroenterol. 2017;112(12):1895-6.

23. Kuo JC, Lilly LB, Hogg D. Immune checkpoint inhibitor therapy in a liver transplant recipient with a rare subtype of melanoma: A case report and literature review. Melanoma Research. 2018;28(1):61-4.

24. Biondani P, De Martin E, Samuel D. Safety of an anti-PD-1 immune checkpoint inhibitor in a liver transplant recipient. Annals of Oncology. 2018;29(1):286-7.

25. Owonikoko TK, Kumar M, Yang S, Kamphorst AO, Pillai RN, Akondy R, et al. Cardiac allograft rejection as a complication of PD-1 checkpoint blockade for cancer immunotherapy: a case report. Cancer Immunol Immunother. 2017;66(1):45-50.

26. Gastman BRE, M. S. Tolerability of immune checkpoint inhibition cancer therapy in a cardiac transplant patient. Ann Oncol. 2016;27(12):2304-5.

27. Qin R, Salama AKS. Report of ipilimumab in a heart transplant patient with metastatic melanoma on tacrolimus. Melanoma Management. 2015;2(4):311-4.
